# suPARnostic: an advanced predictive tool for detecting recurrence in renal cell carcinoma

**DOI:** 10.1186/s12894-023-01337-z

**Published:** 2023-10-24

**Authors:** Nessn Azawi, Karina Sif Sondergaard Mosholt, Nathalie Demuth Fryd, Lars Lund, Juan Ignacio Brignone, Nanna Hvid, Helle Wulf-Johansson, Ole Birger Vesterager Pedersen, Susanne Gjørup Saekmose, Saeed Dabestani

**Affiliations:** 1 Department of Urology, Zealand University Hospital, Sygehusvej 6, Roskilde, 4000 Denmark; 2 Institute of Clinical Medicine, University of Copenhagen, Norregade 10, copenhagen, Copenhagen, 1165 Denmark; 3 Deparment of Urology, Aarhus University Hospital, Palle Juul-Jensens Boulevard 99, 8200 Aarhus N, Aarhus, Denmark; 4 Deparment of Urology, Odense University Hospital, J. B. Winslows Vej 4, Odense, 5000 Denmark; 5 Department of Urology, Aalborg University hospital, Hobrovej 18-22, Aalborg, Aalborg, 9100 Denmark; 6 Department of Clinical Immunology, Naestved Hospital, Ringstedgade 61, Naestved, Naestved, 4700 Denmark; 7 Department of Urology, Kristianstad Central Hospital, Region Skane, Kristianstad, Sweden; 8 Department of Translational Medicine, Division of Urological Cancers, Lund University, Lund, Sweden

**Keywords:** Clear cell kidney Cancer, Biomarker, Liquid biopsy, Prognostication, Recurrence free survival

## Abstract

**Background:**

Plasma soluble urokinase-type Plasminogen Activator Receptor (suPAR) predicts disease aggressiveness in renal cell carcinoma (ccRCC), but its prognostic accuracy has not been investigated. To investigate the prognostic accuracy of preoperative plasma suPAR in patients who received curative treatment for initially localized ccRCC.

**Methods:**

We retrospectively analyzed plasma samples stored in the Danish National Biobank between 2010 and 2015 from 235 patients with ccRCC at any stage. Relationships with outcome analyzed using univariate and multiple logistic Cox regression analysis.

**Results:**

There were 235 patients with ccRCC. The median follow-up period was 7.7 years. In univariate analysis suPAR ≥ 6 ng/mL was significantly associated with overall survival (OS) and recurrence-free survival (RFS). Patients with elevated suPAR were more likely to recur, with a Hazard Ratio (HR) of 2.3 for RFS. In multiple logistic regression, suPAR ≥ 6 ng/mL remained a negative predictor of OS and RFS. Limitations include retrospective study design, wide confidence intervals, and tumor subtype heterogeneity bias.

**Conclusions:**

ccRCC patients with high plasma suPAR concentrations are at an elevated risk of disease recurrence and see lower OS. suPAR is a promising surveillance tool to more precisely follow up with ccRCC patients and detect future recurrences.

**Patient Summary:**

In this study, we showed that new type of liquid marker in blood plasma, called suPAR, is associated to a higher risk of kidney cancer recurrence when elevated above 6ng/mL. We also showed suPAR to independently be able to predict patients overall and recurrence free survival in patient with any stage of kidney cancer.

## Introduction

Renal cell carcinoma (RCC) is the most common malignant kidney tumor and consists of clear cell, papillary, and chromophobe subtypes, with clear cell RCC (ccRCC) accounting for 70–80% of all cases [1]. Annually, approximately 137,000 cases are seen in Europe, 76,000 in North America, and 403,000 globally [2]. The disease is mainly asymptomatic, and at diagnosis, primary metastatic RCC is found in about 15–20% of patients. The remaining patients have localized tumors and are subsequently offered curative treatment to partially or completely remove the affected kidney [3]. An estimated 15–20% of these patients will have a recurrence detected within five years of follow-up [4].

The gold standard postoperative follow-up procedure for recurrence detection is the use of radiological imaging (usually computed tomography (CT) of the chest and abdomen) at regular intervals according to major North American and European guideline recommendations [5]–[8]. In Denmark, standard of care surveillance after curative treatment for RCC is follow-up CT imaging of the thorax and abdomen together with laboratory work-up every six months for two years, and then annually for up to 10 years [9]. Still, approximately 30% of recurrences are found outside of these follow-up protocols. Furthermore, only 10% of patients who go on to develop a recurrence have curable tumors [10]. A recently published study showed that more frequent use of CT imaging for follow-up recurrence detection does not improve post-recurrence survival in patients surgically treated for initially localized RCC [5]. Improved prognostic tools are thus needed to develop more precise surveillance and treatment strategies to increase survival [11].

Newly reported immunological and inflammatory serum biomarkers have shown promise as diagnostic or prognostic tools in RCC [12]–[17]. One novel biomarker in this setting is the soluble urokinase-type Plasminogen Activator Receptor (suPAR), a non-specific marker of systemic inflammation. suPAR is formed by proteolytic cleavage of the membrane-bound urokinase-type Plasminogen Activator Receptor (uPAR) on tumor cells and leukocytes [18]. Upregulation of uPAR proteolysis is seen in various inflammatory and pathological tissue-remodeling processes, including cancer metastasis, fibrinolysis, responses to infectious challenges, and wound healing [19]. It is no surprise, therefore that elevated concentrations of suPAR have been observed in a range of diseases and conditions, including COVID-19, rheumatic disease, cardiovascular disease, acute myocardial infarction, diabetes, pneumonia, sepsis, cancer, and chronic systemic inflammation [20]–[25].

The search for a reliable biomarker in predicting cancer prognosis has led to numerous studies investigating plasma suPAR or uPAR in tumor tissue for improving the diagnosis of cancer, [26] measuring recurrence risk in patients receiving curative surgery, [27], [28] and predicting cancer development in high-risk individuals.[29]. Significant associations have been documented between plasma suPAR or tissue uPAR and detection and survival [26], [30], [31]. Systemic Immune-Inflammation Index has been correlated with disease aggressiveness, survival, and the inflammatory response in RCC [16].

In this study we aimed to investigate the prognostic accuracy of pre-operative plasma suPAR in predicting recurrence and survival in patients who received curative intent treatment for localized ccRCC. Furthermore, we also investigated if suPAR could predict aggressivity of RCC at diagnosis. We hypothesized that an elevated preoperative suPAR would be correlated with poorer overall survival (OS) and recurrence-free survival (RFS).

## Methods

Plasma (100 µl) from 235 patients with pathologically confirmed ccRCC and stored in the Danish National Biobank from five sites in Denmark between January 2010 and December 2015 were identified and used in this study. Only plasma samples collected within 1-180 days before the time of surgery were included. The inclusion criteria required pathologically confirmed ccRCC and availability of stored plasma samples within the specified time frame. Demographic and pathological data were extracted from patients’ electronic medical records after approval by the Danish National Ethical Committee (SJ-836) and the Data Protection Agency (REG-124-2020). All methods were conducted in compliance with relevant guidelines and regulations.

We analyzed the following variables: suPAR level, age, gender, method of treatment, T-stage, Fuhrman grade, Charlson comorbidity index (CCI), presence of hypertension, C-reactive protein (CRP) level, hemoglobin level, and presence of symptoms. Descriptive statistics were presented as mean and standard deviation (SD) or median and interquartile range (IQR). The overall performance of the diagnostic test for suPAR was assessed using the Area Under the Curve (AUC) metric, which measures its accuracy at various thresholds.

suPAR measurements The concentration of suPAR was assessed from plasma using the commercial suPARnostic® assay kit (ELISA, Virogates, Copenhagen, Denmark) and quantified through spectrophotometry, following the manufacturer’s instructions.

Analyses were performed using MedCalc® Statistical Software version 19.6.1 (MedCalc Software Ltd, Ostend, Belgium; https://www.medcalc.org; 2020).

## Results

In total, we included 235 patients with ccRCC in our analyses. The mean age was 66.41 years (SD ± 10.68) and 152 (65%) were male. During the follow-up period, 56 patients died, and 41 patients developed recurrences. There were 151/235 (64%) who underwent radical nephrectomy, 84/235 (36%) who received partial nephrectomy. The median time of follow-up was 7.7 years (IQR 5.18–9.95). The demographic data is presented in (Table 1).

**Table 1:** The demographic data of patients included in study

	Number/Number (SD)	%
Age	65.7 (SD 11.9)	
BMI	27.1(SD 5.9)	
Ischemia time. min	15.1(SD 13.3)	
Operative time. min	153.6 (SD 70.1)	
Tumor size. mm	55.4 (SD 33.4)	
Gender		
Female	84	35.7%
Male	151	64.3%
T stage		
T1	161	68.5%
T2	33	14.0%
T3	41	17.4%
Death	56	23.8%
Surgical techniques		
Partial nephrectomy	84	35.7%
Total nephrectomy	151	64.3%
Fuhrman grad		
1	32	13.6%
2	87	37.0%
3	43	18.3%
4	13	5.6%
Unkown	60	25.5%
Nicrosis	53	22.6%
Symptoms	99	42.1%
Hypertension	84	35.7%
Diabetes_mellitus	35	14.9%
Biopsies	74	31.5%
ASA score		
1	20	8.5%
2	73	31.1%
3	59	25.1%
Unkonw	83	35.3%
Positive lymphnodes	7	3.0%
Smoking		
No	99	42.3%
Yes	111	47.0%
Pervious smokers	25	10.7%

### Associations between preoperative plasma suPAR and outcomes

**Fig. 1:**
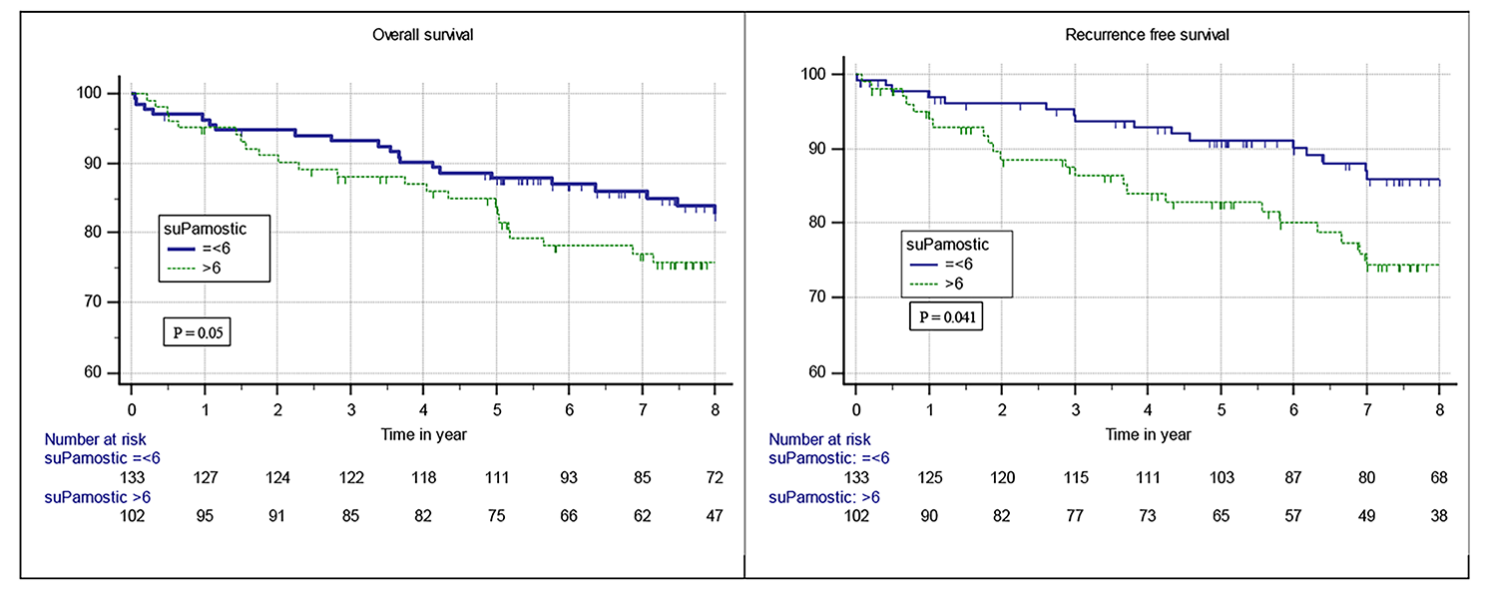
The overall survival and recurrence free survival rate for patients with high and low suPAR, respectively

In univariate analysis, plasma suPAR ≥ 6 ng/ml was a significant negative predictor for both overall survival (HR = 1.69, 95%CI = 0.99–2.89, p = 0.050) and recurrence-free survival (HR = 1.91, 95%CI 1.03–3.57, p = 0.041), Fig. 1. Fuhrman grade of 3 or 4, anaemia, C-reactive protein (CRP) of > 3 mg/L, Charlson Comorbidity Index (CCI) ≥ 3, and advanced tumor T-stage (T3/T4) were also all significant negative predictors of OS; whereas, only Fuhrman grade 3–4 and advanced tumor T-stage were also negative predictors of RFS (Table 2).

**Table 2:** The overall survival and recurrence free survival on univariate analysis

		Overall Survival				Recurrence Free Survival	
	HR	Confidence interval	P value		HR	Confidence interval	P value
**suPARnostic (ng/ml)**			0.0500*				0.00414
< 6	Ref						
≥ 6	1.6893	0.9878 to 2.8890			1.9147	1.0255 to 3.5748	
**CRP (mg/L)**			0.0028*				0.7406
≤ 3	Ref						
> 3	4.2241	1.6415 to 10.8696			1.1799	0.4431 to 3.1419	
**Hemoglobin**			0.0219*				0.7211
Normal or higher	Ref						
Lower than normal	3.7986	1.2134 to 11.8911			0.8179	0.2712 to 2.4663	
**CCI**			< 0.0001*				0.6752
0–2	Ref						
≥ 3	9.7690	4.5078 to 21.1708			1.2334	0.4623 to 3.2907	
**Hypertension**			0.1952				0.0297 *
No	Ref						
yes	1.5397	0.8316 to 2.8508			2.3522	1.0881 to 5.0847	
**Gender**			0.0777*				0.2428
Female	Ref						
Male	1.6260	0.9475 to 2.7904			1.4565	0.7749 to 2.7376	
**Fuhrman grade**			0.0009*				0.0236 *
1–2	Ref						
3–4	3.4564	1.6669 to 7.1672			2.4026	1.1250 to 5.1313	
**T-stage**			0.0022*				< 0.0001*
T1	Ref						
T2	2.3203	0.8580 to 6.2746			2.1881	0.6589 to 7.2660	
T3	2.7059	1.2647 to 5.7894			4.9435	1.9865 to 12.3018	
**Type of surgery**			0.2120				0.0998
Partial Nephrectomy	Ref						
Radical Nephrectomy	2.2837	1.3103 to 3.9800			1.7046	0.9032 to 3.2171	
**Symptomatic at RCC recurrence**			0.2450				0.4084
Asymptomatic	Ref						
Symptomatic	1.3701	0.8058 to 2.3295			0.7703	0.4149 to 1.4301	

Abbreviations: CCI = Charlson Comorbidity Index score, RCC = Renal Cell Carcinoma

In our multiple regression, patients with suPAR levels ≥ 6 ng/ml had a significantly higher risk of mortality compared to those with lower levels (OR = 5.18, 95% CI = 1.50–17.93, p = 0.009) and patients with a CCI score ≥ 3 had a significantly higher risk of mortality compared to those with a lower score (OR = 2.74, 95%CI = 1.38–5.43, p = 0.004). Each unit increase in age was associated with a slight increase in the risk of mortality, but the association was not statistically significant (OR = 1.07, 95% CI = 0.99 to 1.15, p = 0.1083). Fuhrman grade (3 or 4) was associated with an increased risk of mortality, but the association was not statistically significant (OR = 3.47, 95% CI = 0.96 to 12.58, p = 0.058).

Patients with suPAR levels ≥ 6 ng/ml had a higher risk of recurrence compared to those with lower levels (OR = 2.29, 95% CI = 1.02 to 5.15, p = 0.0458) and patients with advanced tumor T-stage (T2 or T3) had a significantly higher risk of recurrence compared to those with T1 stage (OR = 2.02, 95% CI = 1.22 to 3.31, p = 0.0055), when adjusting for Fuhrman grade, presence of symptoms at time of recurrence, type of treatment, and gender (AUC 0.75, 95%CI; 0.683 to 0.815) (Table 3). Higher Fuhrman grade was not significantly associated with the risk of recurrence (OR = 1.19, 95% CI = 0.71 to 1.98, p = 0.5063).

**Table 3:** The overall survival and recurrence free survival on multivariate analyses

**Overall Survival**			
**Variable**	**Odds ratio**	**95% CI**	**P value**
**suPARnostic**	5.1845	1.4987 to 17.9356	0.0094*
**CCI**	2.742	1.3821 to 5.4397	0.0039*
**Age**	1.0657	0.9861 to 1.1517	0.1083
**Symptomatic at diagnosis**	0.4789	0.1390 to 1.6499	0.2434
**Type of Treatment**	0.3538	0.0737 to 1.6993	0.1944
**Male gender**	2.4869	0.7034 to 8.7927	0.1574
**T-stage**	2.6858	1.1148 to 6.4705	0.0276
**Fuhrman grade**	3.4722	0.9586 to 12.5772	0.058
**Recurrence Free Survival**			
**suPARnostic**	2.2864	1.0156 to 5.1470	0.0458*
**T-stage**	2.0156	1.2284 to 3.3073	0.0055*
**Fuhrman grade**	1.189	0.7137 to 1.9808	0.5063
**Type of Treatment**	1.3676	0.5279 to 3.5430	0.5192
**Male gender**	2.106	0.8447 to 5.2507	0.1101

Abbreviations: CCI = Charlson Comorbidity Index score, RCC = Renal Cell Carcinoma

### Prognostic performance of suPAR compared to the Leibovich score system (2003 model)

**Fig. 2:**
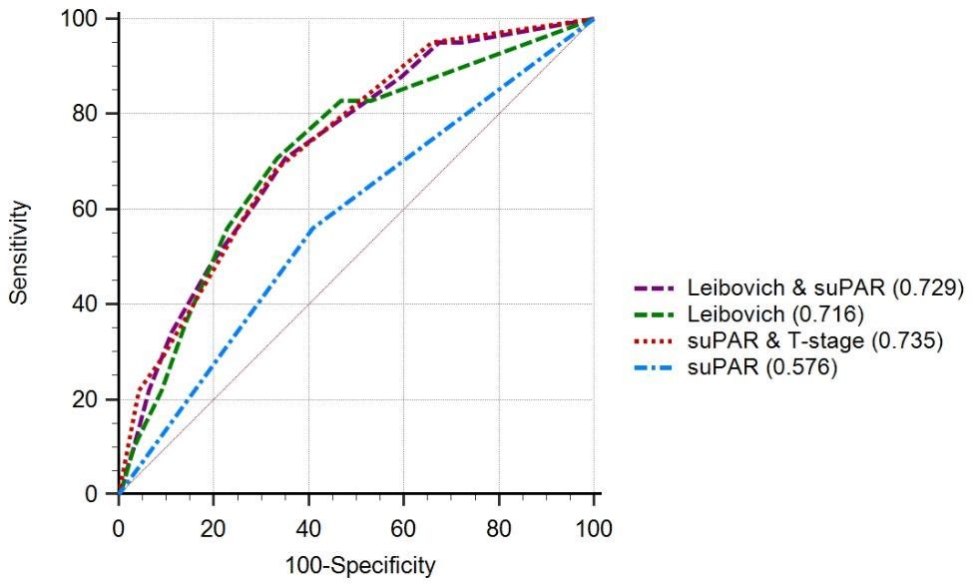
The overall comparison between different nomograms and variables

The prognostic performance for suPAR was 0.576, 95%CI = 0,509–0,641. The prognostic performance of the Leibovich score system (2003 model) in this cohort was as follows: AUC = 0.719, 95%CI = 0.657–0.775. Adding the suPAR measurements as an additional variable to the Leibovich score system did not significantly improve the diagnostic accuracy of the Leibovich Scoring system (AUC = 0.736, 95%CI = 0.675–0.791, p = 0.3468). Additionally, the combination of suPAR and T-stage provided the same diagnostic performance as the Leibovich score system alone (AUC = 0.735, 95% CI = 0.673–0.791, p = 0.5195) (Fig. 2).

## Discussion

This retrospective study investigated the prognostic value of a potentially novel plasma-based biomarker for RCC. Indeed, suPAR has to the best of our knowledge not previously been investigated as a prognostic tool for RCC as it has for other cancers [32]. We were able to show that preoperative plasma suPAR levels were as good as Leibovich score in predicting disease recurrence but also that suPAR was an independent prognostic marker for OS and RFS. The clear benefit of suPAR is that it is plasma-based, allowing for easier triage and reduced healthcare costs. suPAR predicting the recurrences before the surgery can be a useful tool for identifying patients for adjuvant immunotherapy in the future. Our study did not investigate the diagnostic accuracy of suPAR and further studies would be needed.

Previous biomarker studies have found associations between potential plasma-based biomarkers for RCC and survival, but few have compared their performance to widely used prognostic tools. One measure of systemic inflammation, the systemic immune-inflammation index (SII), has been associated with lower OS in RCC (p < 0.001) but not with disease-free survival in a meta-analysis that included ten studies [16]. SII was however associated with disease aggressiveness, in line with our study. Another inflammatory biomarker, pre-operative c-reactive protein (CRP), was found to be an independent prognostic marker for RCC in univariate analysis for both OS as well as RFS, however, we only found CRP to predict OS in univariate analysis [14]. These studies concur with ours in that systemic inflammation speeds up any potential RCC progression after curative surgery and can therefore be used as a prognostic tool.

There is a scarcity of literature on plasma suPAR as a prognostic marker in cancer, let alone in RCC, but the few studies which have investigated the prognostic utility of suPAR have found significant negative associations between elevated levels and any survival-related outcomes. In colorectal cancer, suPAR above a median of 4.25 ng/ml was negatively associated with OS (p = 0.01) but not with progression-free survival (PFS) [25]. Our study used a higher cut-off value for suPAR and found significant associations with both OS and RFS, although, no standardized or biological cut off level for plasma suPAR has been established for recurrence detection, nor was any such level determined in our current investigation.

Previous research investigating the role of the plasminogen activation system on cancer progression has measured uPAR expression on excised tumor tissue, blood levels of urokinase-type plasminogen activator (uPA), and the various forms of uPAR and suPAR in circulation. There is currently no consensus on which method is most prognostically accurate, but plasma suPAR has been studied more recently, showing promise as a stable biomarker [24]. In urothelial carcinoma (UC), Dohn et al., showed that excised tumor tissue that highly expressed uPAR was significantly associated with a lower OS and RFS [30]. They later found that preoperative elevated plasma suPAR, as in our study, also predicted lower recurrence-free survival (HR = 7.55, 95%CI = 2.03–28.03, p = 0.003) and overall survival (HR = 2.11, 95%CI = 1.35–3.11, p = 0.001) in multivariate analysis in patients with UC [27]. Unlike our study, they evaluated patients prospectively who were to undergo radical cystectomy and aggregated various plasma suPAR forms using fluorescence immunoassays, whereas we used an ELISA assay to quantify total circulating suPAR.

Compared to uPAR, suPAR requires fewer steps to measure and is thus more accessible clinically. Measuring cell-surface uPAR requires several steps, including freezing tumor tissue and preparing an extract, whereas suPAR can be measured non-invasively. Furthermore, suPAR can be detected in healthy individuals and patients with RCC, providing greater utility. Additionally, uPAR is not expressed uniformly across cell types or cancer cells as it has been found to be most highly expressed in cancer cells located on the invasive front of bladder tumors compared to cells located within the tumor core in the study by Dohn et al., with myofibroblasts having the greatest expression rates among cell types, as measured by immunohistochemistry [30]. Nevertheless, our study adds to the body of evidence illuminating the role of the plasminogen activating system in facilitating the spread and growth of RCC.

Our study also compared suPAR with the 2003 Leibovich scoring system and found suPAR as good as Leibovich score as a prognostic tool when paired with T-stage. Sim et al., similarly compared the prognostic performance of RCC biomarkers with previously published nomograms, [14] but not the Leibovich scoring system, which is to date the most prognostically accurate nomogram in clinical use for predicting recurrence risk for curatively treated localized RCC [33]. Their study found that pre-operative plasma osteopontin, carbonic anhydrase IX, and CRP together, outperformed the Karakiewicz’s nomogram [34]. Unlike our study, none of the three biomarkers in Sim et al., could accurately predict RCC prognosis alone when used with other standard prognostic factors. The main benefit of suPAR over Leibovich score is that it can be used as a pre-operative test, whereas the Leibovich score is based on on a combination of clinical and post-operative pathological factors.

Detecting patients at high risk for RCC recurrence after curative treatment remains an unmet medical need. Many nomograms fail to this end, either due to the complexity of the nomogram or the low accuracy rate [35], [36]. Numerous biomarkers for RCC have demonstrated promising results in predicting recurrence-free survival, yet none have been validated and standardized for clinical use. suPAR as a single biomarker is independently associated with OS and RFS, yet, a combination approach involving multiple biomarkers may improve the prognostic accuracy even further and remains to be investigated.

Currently, no biomarker is being used to detect disease progression in patients with RCC, with imaging still being the mainstay option. suPAR is believed to facilitate the spread of cancer cells through various mechanisms, including degrading and dissolving extracellular matrix (ECM) by activating the plasminogen activation system and promoting chemotaxis [18], [37]–[40]. Unlike other markers of inflammation, such as c-reactive protein (CRP), suPAR is less sensitive to changes in health and may be a more stable prognostic marker [41]. Despite its stability, plasma suPAR concentrations have been found to decline following chemotherapy [42].

The strengths of this study are primarily that several highly clinically and histologically relevant co-variates were adjusted for in a multiple regression model. Additionally, the prognostic accuracy of suPAR concentrations was found to be non-inferior to the 2003 Leibovich score. The diagnostic accuracy of suPAR could not be investigated in our study due to its retrospective nature and the finding of RCC tumors that were concurrent with other malignancies as identified in histological analysis. The study’s limitations include small study cohortproviding wide confidence intervals, tumor subtype heterogeneity bias, and the retrospective nature of the study design. More studies are needed to compare plasma suPAR levels in patients with RCC with that of healthy controls in order to evaluate its predictive value.

## Conclusion

This study highlights the potential of measuring suPAR as a predictive tool in RCC progression, with elevated suPAR levels (> 6 ng/ml) indicating a two-fold increase in recurrence risk. Adjusting for relevant clinical and histological parameters, preoperative plasma suPAR shows promise as a prognostic indicator for recurrence and overall survival in RCC. Further validation and standardization are needed to establish suPAR as a liquid biomarker for RCC.

## Data Availability

The datasets produced and/or examined during the course of this study are not accessible to the public, as they contain sensitive information that could potentially identify patients. In accordance with the guidelines set forth by the ethical committee and data protection agency, the publication of any such data is strictly prohibited. Please contact: nesa@regionsjealland.dk.
